# Assessment of pharmacokinetics-pharmacodynamics to support omadacycline dosing regimens for the treatment of patients with acute bacterial skin and skin structure infections

**DOI:** 10.1128/aac.01281-23

**Published:** 2024-07-31

**Authors:** Sujata M. Bhavnani, Jeffrey P. Hammel, Elizabeth A. Lakota, Kathryn Liolios, Michael Trang, Christopher M. Rubino, Judith N. Steenbergen, Lawrence Friedrich, Evan Tzanis, Paul G. Ambrose

**Affiliations:** 1 Institute for Clinical Pharmacodynamics, Inc., Schenectady, New York, USA; 2 Paratek Pharmaceuticals, Inc., King of Prussia, Pennsylvania, USA; Providence Portland Medical Center, Portland, Oregon, USA

**Keywords:** acute bacterial skin and skin structure infections, pharmacokinetics-pharmacodynamics, omadacycline

## Abstract

Pharmacokinetic-pharmacodynamic (PK-PD) relationships for efficacy were evaluated using data from omadacycline-treated patients with acute bacterial skin and skin structure infections (ABSSSI) enrolled in two phase 3 studies. Patients received omadacycline 100 mg intravenously (IV) every 12 hours for two doses, followed by 100 mg IV every 24 hours (q24h), with the option to switch to 300 mg oral (PO) q24h after 3 days or 450 mg PO q24h for two doses, followed by 300 mg PO q24h for a total duration of 7–14 days. Clinical response was evaluated at 48–72 hours [early clinical response (ECR)], end of treatment (EOT), and 7–14 days after EOT. Using a population pharmacokinetic (PK) model and PK data from patients with *Staphylococcus aureus* at baseline, omadacycline free-drug plasma area under the concentration-time curve (AUC) values were determined, and the relationships between free-drug plasma AUC:MIC ratio and dichotomous efficacy endpoints were evaluated. Using these relationships, the population PK model, simulation, and an omadacycline MIC distribution for *S. aureus*, mean percent probabilities of response were evaluated. Statistically significant PK-­PD relationships were identified for ECR (*P* = 0.016 and 0.013 for optimized two- and three-group free-drug plasma AUC:MIC ratios, respectively). At an MIC value of 0.5 µg/mL, percent probabilities of model-predicted success for ECR based on the univariable PK-PD relationships using continuous and two-group free-drug plasma AUC:MIC ratio variables were 91.9 and 95.6%, respectively, for the IV-to-PO dosing regimen and 89.3 and 88.4%, respectively, for the PO-only dosing regimen. These data support for omadacycline IV-to-PO and PO-only dosing regimens for ABSSSI and an omadacycline susceptibility breakpoint of 0.5 µg/mL for *S. aureus*.

## INTRODUCTION

Omadacycline, an aminomethylcycline that is structurally related to tetracycline agents, demonstrates *in vitro* activity against pathogens commonly associated with acute bacterial skin and skin structure infections (ABSSSI), including *Staphylococcus aureus*, methicillin-resistant isolates, and beta-hemolytic streptococci (1, 2). Omadacycline intravenous (IV) and oral (PO) formulations were approved in October 2018 by the United States Food and Drug Administration (US FDA) for the treatment of adult patients with ABSSSI and community-acquired bacterial pneumonia (CABP) (3). As described below, late-stage pharmacometric analyses were carried out to provide support to omadacycline IV-to-PO and PO-only dosing regimens for the treatment of patients with ABSSSI.

The evaluation of pharmacokinetic-pharmacodynamic (PK-PD) relationships for efficacy based on clinical data collected in phase 3 and the results of PK-PD target attainment analyses carried out using a population pharmacokinetic (PK) model developed using phase 1 and 3 data provide valuable information to confirm early-stage dose selection decisions (4, 5). Guidance for Industry from the US FDA (6) and that from the European Medicines Agency (7) also describe the benefit of evaluating PK and PK-PD data during late-stage development. The collection of omadacycline PK data in phase 3 clinical studies (8–11) proved useful to refine a previously developed population PK model describing the disposition of omadacycline that was originally constructed using phase 1 data (12, 13). Covariate analyses using the final population PK data were performed and supported the determination that dose adjustment on the basis of patient factors was not needed (14, 15).

PK data from two phase 3 pivotal studies for ABSSSI, OASIS-1 and OASIS-2 (10, 11), were used along with the above-described population PK model (13) to estimate exposures in individual omadacycline-treated patients from these studies. Patients enrolled in the OASIS-1 study received IV omadacycline or IV linezolid, with the option to switch to PO formulations after 3 days if there was evidence of clinical improvement, while patients enrolled in the OASIS-2 study received PO-only formulations of omadacycline or linezolid. As described herein, these data provided the opportunity to evaluate the PK-PD relationships for omadacycline efficacy in patients with ABSSSI. Additionally, using the population PK model (13) and non-clinical PK-PD targets for efficacy (16), PK-PD target attainment analyses were carried out to provide support for omadacycline dosing regimens for the treatment of patients with ABSSSI and interpretive criteria for the *in vitro* susceptibility testing of omadacycline against *S. aureus*.

## RESULTS

### PK-PD analyses for efficacy

A total of 182 omadacycline-treated patients with ABSSSI had sufficient PK data, an appropriate source pathogen, and MIC data, and were evaluable for at least one efficacy endpoint in the pooled OASIS-1 and OASIS-2 microbiologically evaluable (ME) populations at the end-of-treatment visit (EOT) (ME-EOT) or ME post-treatment evaluation visit (PTE) (ME-PTE). Of these patients, 128 had *S. aureus* at baseline. Summary statistics for categorical and continuous patient characteristics for these 128 patients are provided by study and pooled in Tables S1 and S2, respectively. Patients in the OASIS-2 study were generally younger (median age, 42 vs 51 years), more frequently had a wound infection (72.2 vs 12.9%), less frequently had cellulitis/erysipelas (11.3 vs 51.6%), more often had polymicrobial infections (37.1 vs 19.4%), more often were patients who injected drugs or had infection due to injection drug use (73.2 vs 29.0%), and generally had higher baseline creatinine clearance (median, 104 vs 79.5 mL/min/1.73 m^2^).

A summary of successful responses for efficacy endpoints by visit for the above-described patients with *S. aureus* at baseline is shown in Table S3. The percentage of patients with success was 92.8% for early clinical response (ECR) assessed at 48–72 hours following initiation of therapy and ranged from 99.1 to 99.2% among the clinical and microbiological response endpoints assessed at EOT and PTE. The summary of dichotomous lesion area reduction endpoints by day or visit, which ranged from cessation to ≥70% reduction from baseline, is shown in Table S4. While the percentage of patients achieving these endpoints was lower on day 2 relative to subsequent study days or visits, a high percentage of patients achieved endpoints up to ≥30% reduction in lesion area from baseline by day 3. By day 5, 83.9% had achieved ≥ 50% reduction from baseline and by day 7, 84.1% had achieved ≥ 70% reduction from baseline. Summary statistics for omadacycline free-drug plasma area under the concentration-time curve (AUC), baseline MIC, and free-drug plasma AUC:MIC ratio for these patients are provided by study and pooled in Table S5. The distributions of observed baseline omadacycline MIC values for the two studies were similar. Mean and median values for the 24-hour average free-drug plasma AUC values over the first 48 hours and free-drug plasma AUC:MIC ratios based on these AUC values for patients from the OASIS-2 study were a little more than half of those for patients from the OASIS-1 study.

A summary of *P*-values and directions for univariable relationships between the probability of achieving dichotomous clinical or microbiological response endpoints and omadacycline free-drug plasma AUC:MIC ratio evaluated as continuous or categorical variables is shown in Table S6. Of these univariable relationships, there were a limited number of statistically significant or borderline significant relationships identified, each of which were for ECR. While the data for the univariable relationships for dichotomous lesion size endpoints were supportive of the relationships between free-drug plasma AUC:MIC ratio and the probability of success for ECR that were identified, the results of the univariable analyses for the time-to-event and continuous lesion size endpoints did not yield informative relationships (data not shown).

**Fig 1:**
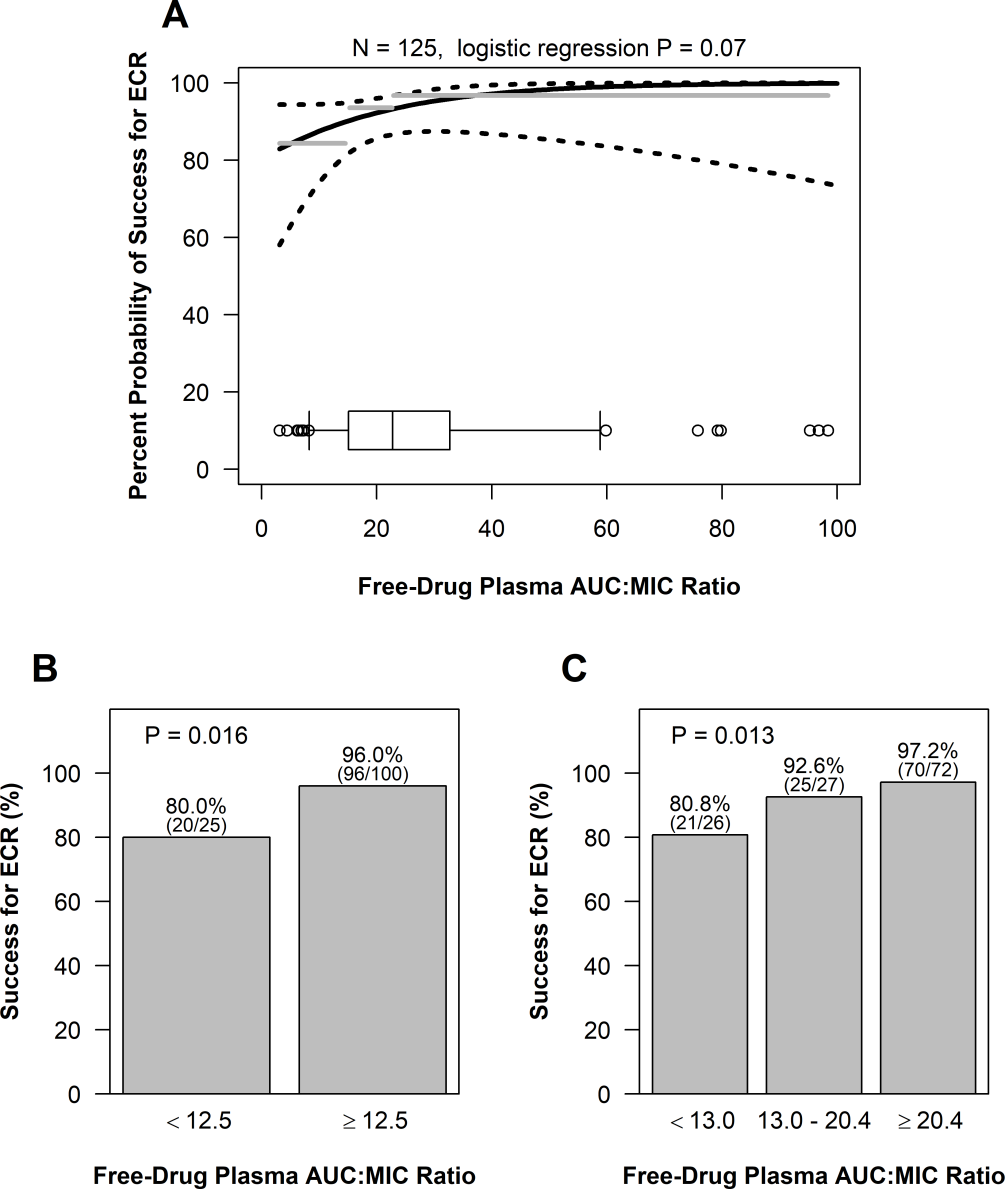
Univariable relationships between the percent probability of success for ECR and free-drug plasma AUC:MIC ratio evaluated as continuous (**A**), two-group (**B**), and three-group (**C**) variables based on data from patients with ABSSSI and *S. aureus* at baseline. The dashed lines in panel A represent 95% pointwise confidence bands on the fit of the logit function. The gray line segments show the observed percentage of patients with success for ECR within each quartile of the free-drug plasma AUC:MIC ratio. The boxplot at the bottom of the figure shows the distribution of the free-drug plasma AUC:MIC ratio. The box portion covers the 25^th^ to 75^th^ percentiles, with a central line at the median, with whiskers drawn to the 5^th^ and 95^th^ percentiles, and with observations outside those extremes individually shown by circles.

Panels A, B, and C of Fig. 1 show the univariable relationships between the probability of success for ECR and each of free-drug plasma AUC:MIC ratio evaluated as continuous, two-group, and three-group variables, respectively, based on data from patients with *S. aureus*. While not demonstrating statistical significance at the 0.05 level, there was evidence of a univariable relationship between the probability of success for ECR and free-drug plasma AUC:MIC ratio evaluated as a continuous variable (*P* = 0.07) as shown in panel A of Fig. 1.

The univariable relationships between the probability of success for ECR and free-drug plasma AUC:MIC ratio evaluated as two-group and three-group variables shown in panels B and C of Fig. 1, respectively, were of greater statistical significance (*P* = 0.016 and 0.013, respectively) than that for the above-described relationship shown in panel A. The free-drug plasma AUC:MIC ratio thresholds for the lower group for these two relationships were almost identical (12.5 and 13.0, respectively), differing by just one patient. For the three-group variable, the percentage of patients with success for ECR for the middle free-drug plasma AUC:MIC ratio group of 92.6% was closer to 97.2% for the highest AUC:MIC ratio group than 80.8% for the lowest AUC:MIC ratio group. Thus, the univariable relationships for the two- and three-group free-drug plasma AUC:MIC ratio variables were, therefore, similar in both appearance and statistical significance.

A 95% bootstrap confidence interval for the above-described two-group free-drug plasma AUC:MIC ratio threshold of 12.5 ranged from 11.6 to 22.9. Based on the logistic regression model for the assessment of free-drug plasma AUC:MIC ratio evaluated as a continuous variable, the estimated probability of success for ECR [95% confidence interval (CI)] at the threshold of 12.5 was 88.9% (78.4–94.6%). The estimated probabilities of success for ECR (95% CI) at the magnitude of the non-clinical free-drug plasma AUC:MIC ratio targets for net bacterial stasis and 0.5- and 1-log_10_ CFU reductions were 93.0% (86.4–96.5%), 96.4% (87.2–99.1%), and 98.9% (84.0–99.9%), respectively.

Evaluations of relationships with free-drug plasma AUC and MIC individually were also carried out (data not shown). Given that 68.8% of patients had an isolate with an MIC value of 0.25 µg/mL and all of the remaining patients had an isolate with an MIC value of 0.12 or 0.5 µg/mL, there was insufficient variability in MIC values to detect any relationships with this variable. As a result of the limited variability in MIC values, relationships with free-drug plasma AUC generally mimicked the relationships with the free-drug plasma AUC:MIC ratio. Thus, given these findings, greater focus was given to the univariable relationships identified between free-drug plasma AUC:MIC ratio and the probability of success for ECR. However, given the limited the number of failures observed for the ECR endpoint, multivariable logistic regression analyses were not performed.

### Evaluation of PK-PD target attainment and model-predicted efficacy among simulated patients

**Fig 2:**
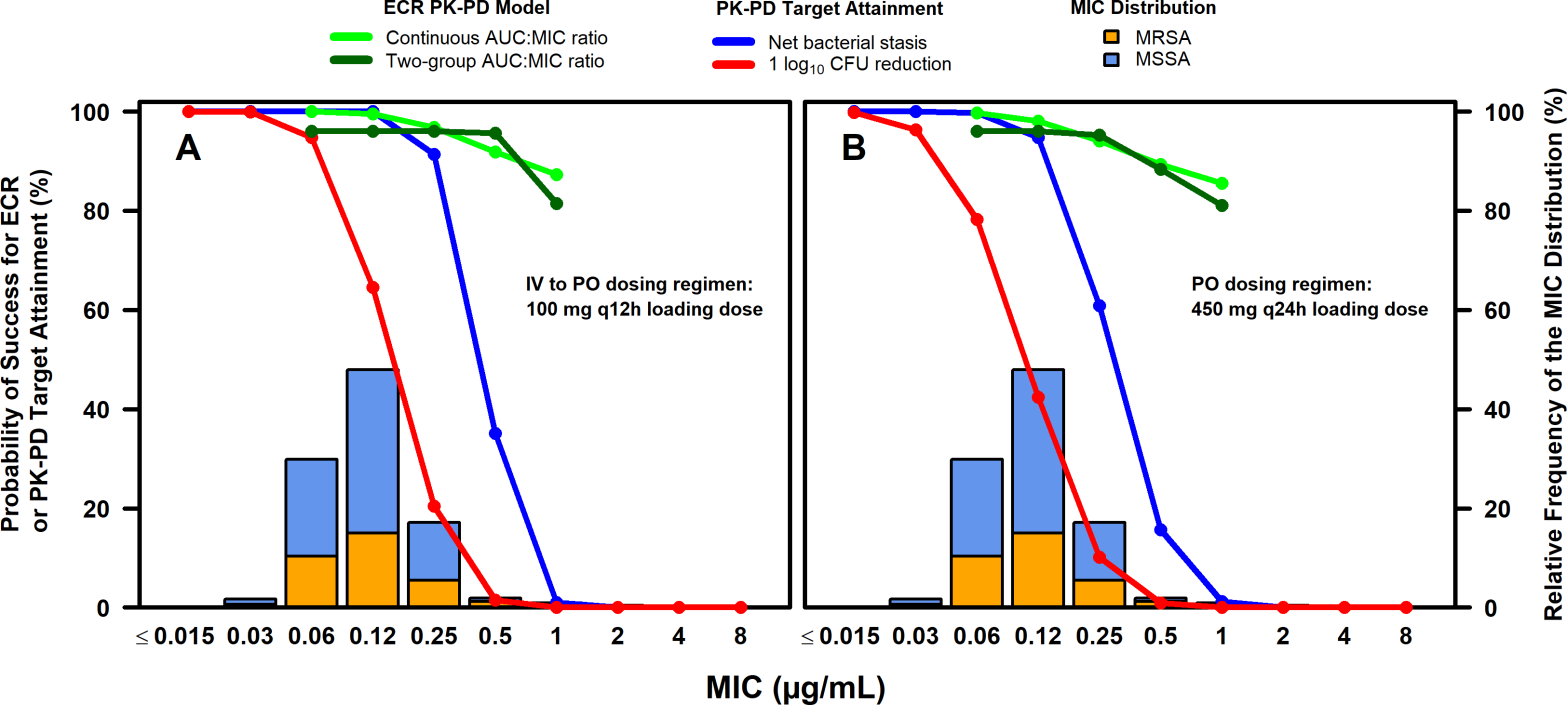
Percent probabilities of PK-PD target attainment on days 1–2 and model-predicted success for ECR by MIC among simulated patients after administration of omadacycline IV-to-PO with 100 mg IV q12h loading dose (**A**) and PO-only (**B**) dosing regimens, overlaid on MIC distribution for *S. aureus* isolates from the USA and Europe.

Percent probabilities of PK-PD target attainment on days 1–2 and model-predicted success for ECR by MIC are shown overlaid on the MIC distribution for *S. aureus* isolates collected from medical centers in the USA and Europe for the omadacycline IV-to-PO (panel A) and the PO-only (panel B) dosing regimens in Fig. 2. The stacked MIC bars in each figure panel show the proportion of *S. aureus* isolates that were susceptible or ­resistant to methicillin. Model-predicted success for ECR by MIC is only shown for the two-group assessment given that the relationships between the probability of success for ECR and free-drug plasma AUC:MIC ratio when evaluated as either a two- or three-group variable were similar. The percent probabilities of PK-PD target attainment on days 1–2 and model-predicted success for ECR by selected MIC among simulated patients after the administration of the omadacycline IV-to-PO dosing regimen containing the 100 mg IV every 12 hours (q12h) loading dose on day 1 and the PO-only dosing regimen are also shown in Table 1. The observed percentage of success for ECR and clinical responses at PTE by MIC among omadacycline-treated patients with ABSSSI and *S. aureus* at baseline from the pooled phase 3 OASIS-1 and OASIS-2 studies (10, 11) is also shown in Table 1. Observed outcomes did not demonstrate discernable trends with increased or decreased MIC in the microbiological modified intent-to-treat (micro-mITT) and ME populations inclusive of patients without PK data as shown in Table 1.

**TABLE 1:** Comparison of observed percentages of successful clinical response by MIC among patients with ABSSSI and *S. aureus* at baseline from the pooled phase 3 OASIS-1 and OASIS-2 studies and percent probabilities of PK-PD target attainment on days 1–2 and model-predicted success for ECR by MIC among simulated patients after the administration of omadacycline IV-to-PO or PO-only dosing regimens

MIC (µg/mL)	Observed percentage of successful clinical response by MIC by study population^*a*^				Percent probability of PK-PD target attainment on days 1–2 and model-predicted success for ECR at 48–72 hours for two univariable PK-PD relationships by MIC among simulated patients by omadacycline dosing regimen^*b*^^,*c*^					
					Assessment of IV-to-PO dosing regimen^*d*^			Assessment of PO-only dosing regimen^*e*^		
	micro-mITT population		ME populations		PK-PD target attainment^*f*^	Univariable PK-PD relationships		PK-PD target attainment^*f*^	Univariable PK-PD relationships	
	ECR at 48–72 hours (*N* = 365)	Clinical response at PTE (*N* = 365)	ECR at 48–72 hours (*N* = 339)	Clinical response at PTE (*N* = 291)		Continuous	Two-group		Continuous	Two-group
0.06	0	0	0	0	100	100	96.0	99.7	99.7	96.0
0.12	92.7 (38/41)	80.5 (33/41)	97.4 (38/39)	100 (33/33)	100	99.5	96.0	94.8	98.1	96.0
0.25	86.5 (225/260)	81.9 (213/260)	93.3 (224/240)	97.5 (199/204)	91.3	96.8	96.0	60.8	94.0	95.2
0.5	91.9 (57/62)	90.3 (56/62)	96.6 (56/58)	100 (53/53)	35.1	91.9	95.6	15.7	89.3	88.4
1	100 (2/2)	100 (2/2)	100 (2/2)	100 (1/1)	1.04	87.2	81.5	1.24	85.6	81.1
Overall^*g*^	88.2 (322/365)	83.3 (304/365)	94.4 (320/339)	98.3 (286/291)						
All					95.8	98.9	95.8	87.7	97.5	95.5
MRSA					92.3	98.4	95.4	84.3	97.1	95.0
MSSA					97.7	99.1	96.0	89.4	97.8	95.8

^*a*^ Based on data from patients with ABSSSI and *S. aureus* at baseline in the micro-mITT and ME populations of the OASIS-1 and OASIS-2 studies (10, 11).

^*b*^ Assessed using free-drug plasma AUC:MIC ratio targets associated with a net bacterial stasis CFU reduction from baseline for *S. aureus* based on data from a neutropenic murine-thigh infection model (16).

^*c*^ Based on the assessment of average free-drug plasma AUC_0–24_ values on days 1 and 2.

^*d*^ Omadacycline 100 mg IV q12h on day 1, followed by 100 mg IV q24h on day 2 with a PO switch to 300 mg PO q24h on days 3–5.

^*e*^ Omadacycline 450 mg PO q24h on days 1 and 2, followed by 300 mg PO q24h on days 3–5.

^*f*^ Using data for all *S. aureus* isolates studied, free-drug plasma AUC:MIC ratio targets associated with net bacterial stasis were randomly assigned based on an estimated log normal distribution of AUC:MIC ratio targets associated with the same endpoint.

^*g*^ Overall represents the percentage of successful clinical response among all observed patients or the percent probability of PK-PD target attainment weighted over the given MIC distribution (1) for simulated patients.

At the MIC value of 0.25 µg/mL (i.e., the MIC value at which 90% of isolates were inhibited), percent probabilities of PK-PD target attainment on days 1–2 based on randomly assigned free-drug plasma AUC:MIC ratio targets associated with net bacterial stasis were 91.3 and 60.8% for the IV-to-PO and PO-only dosing regimens, respectively. Percent probabilities of success for ECR at an MIC value of 0.25 µg/mL for univariable relationships based on the continuous and two-group free-drug plasma AUC:MIC ratio variables were 96.8 and 96.0%, respectively, for the IV-to-PO dosing regimen and 94.0 and 95.2%, respectively, for the PO-only dosing regimen. Corresponding 95% lower bootstrap confidence bounds for percent probabilities of success for ECR for the two dosing regimens based on the free-drug plasma AUC:MIC ratio evaluated as a continuous variable were 90.7 and 88.9%, respectively.

At an MIC value of 0.5 µg/mL, percent probabilities of PK-PD target attainment decreased to 35.1 and 15.7% for the IV-to-PO and PO-only dosing regimens, respectively. However, percent probabilities of success for ECR at an MIC value of 0.5 µg/mL for univariable relationships based on the continuous and two-group free-drug plasma AUC:MIC ratio variables were 91.9 and 95.6%, respectively, for the IV-to-PO dosing regimen and 89.3 and 88.4%, respectively, for the PO-only dosing regimen. Corresponding 95% lower bootstrap confidence bounds for percent probabilities of success for ECR for the two dosing regimens based on the free-drug plasma AUC:MIC ratio evaluated as a continuous variable were 84.7 and 79.1%, respectively.

At an MIC value of 1 µg/mL, percent probabilities of success for ECR for univariable relationships based on free-drug plasma AUC:MIC ratio evaluated as continuous and two-group variables were 87.2 and 81.5%, respectively, for the IV-to-PO dosing regimen and 85.6 and 81.1%, respectively, for the PO-only dosing regimen. The corresponding 95% lower bootstrap confidence bounds for percent probabilities of success for ECR at an MIC value of 1 µg/mL based on free-drug plasma AUC:MIC ratio evaluated as a continuous variable were 70.9 and 61.0% for the IV-to-PO and PO-only dosing regimens, respectively. Therefore, for an MIC value of 1 µg/mL, it could not be established with high confidence that the percent probability of success for ECR was at least 80%.

Across the MIC distribution for all isolates including the methicillin-resistant *S. aureus* and methicillin-susceptible *S. aureus* subsets, overall percent probabilities of PK-PD target attainment on days 1–2 for the two dosing regimens ranged from 84.3 to 97.7%. Overall percent probabilities of model-predicted success for ECR ranged from 95.0 to 99.1% for the two dosing regimens and free-drug plasma AUC:MIC ratio as either a continuous or two-group variable.

The above-described percent probabilities of PK-PD target attainment on days 1–2 and model-predicted success for ECR by MIC among simulated patients after administration of the omadacycline IV-to-PO dosing regimen containing the 200 mg IV every 24 hours (q24h) loading dose on day 1 are shown in Fig. S1 and provided in Table S7. These results are shown compared to those for the omadacycline IV-to-PO dosing regimen containing the 100 mg IV q12h loading dose on day 1. The similar findings across the two IV-to-PO dosing regimens were expected given that the PK-PD index most closely associated with efficacy for tetracyclines is the AUC:MIC ratio (17–20) and that the day 1 total omadacycline dose was the same, and thus, AUC was nearly identical.

## DISCUSSION

The analyses described herein were carried out to provide support for the use of IV-to-PO and PO-only omadacycline dosing regimens for the treatment of patients with ABSSSI. The specific objectives were twofold. The first objective was to evaluate PK-PD relationships for efficacy based on pooled data from omadacycline-treated patients with ABSSSI enrolled in the OASIS-1 (IV-to-PO) and OASIS-2 (PO-only) studies. The second objective was to use identified clinical PK-PD relationships based on these analyses and non-clinical PK-PD targets for efficacy, together with Monte Carlo simulation, to evaluate omadacycline IV-to-PO and PO-only dosing regimens. Results of the latter set of analyses were also used to evaluate interpretive criteria for the *in vitro* susceptibility testing of omadacycline against *S. aureus*. As described below, results of both sets of analyses provide support for omadacycline dose selection for patients with ABSSSI.

The PK-PD analyses for efficacy were based on data from 128 omadacycline-treated patients with ABSSSI and sufficient PK data in the ME populations of each study, and with *S. aureus* at baseline. While this number of patients was robust, data based on the collection of serial lesion size data over days 1–7 allowed for a richer data set and, accordingly, time-to-event analyses. While univariable PK-PD analyses for efficacy endpoints at EOT or PTE were not identified due to a limited number of observed failures, PK-PD relationships for ECR were evident and are described below. Since the number of observed patients with failure was also limited for ECR, it was, however, not possible to assess the impact of other independent variables that may have impacted the probability of clinical success for this endpoint.

As free-drug plasma AUC:MIC ratio evaluated as a continuous variable increased, so too did the probability of success for ECR (*P* = 0.07). At a free-drug plasma AUC:MIC ratio that approached 0, the percent probability of success for ECR was approximately 80%. At a free-drug plasma AUC:MIC ratio of 14.7, the percent probability of success for ECR was 90%. PK-PD relationships for ECR of greater statistical significance were also evident when free-drug plasma AUC:MIC ratio was evaluated as two- and three-group variables (*P* = 0.016 and 0.013, respectively). For the former relationship, the percentage of patients with success for ECR was 80% (20/25) and 96.0% (96/100) for free-drug plasma AUC:MIC ratio <12.5 and ≥12.5, respectively. The majority of patients (80%, 100/125) had free-drug plasma AUC:MIC ratios equal to or above 12.5.

The 95% bootstrap confidence interval for the two-group free-drug plasma AUC:MIC ratio threshold of 12.5 ranged from 11.6 to 22.9 and included the non-clinical free-drug plasma AUC:MIC ratio target associated with net bacterial stasis of 21.9, which was derived from data from a neutropenic murine-thigh infection model (16). The logistic regression model for free-drug plasma AUC:MIC ratio evaluated as a continuous variable predicted 88.9 and 93.0% probabilities of success for ECR at the thresholds of 12.5 and 21.9, respectively. The median free-drug plasma AUC:MIC ratio target associated with a 1-log_10_ CFU reduction from baseline, 57.7, was outside the above-described 95% bootstrap confidence interval. These findings suggest that achieving a non-clinical PK-PD target associated with bacterial reductions greater than net bacterial stasis is not warranted for optimal efficacy in patient populations resembling those studied. The implication of these findings is that the use of non-clinical PK-PD targets associated with greater reductions in bacterial burden for drug development dose selection decisions for the treatment of patients with ABSSSI may lead to selection of higher doses than needed and, thus, unnecessary exposure-related toxicity.

The PK-PD relationships for ECR identified among omadacycline-treated patients with ABSSSI and *S. aureus* at baseline were used to assess model-predicted success for ECR by MIC among simulated patients. Percent probabilities of model-predicted success for ECR were compared to percent probabilities of achieving a free-drug plasma AUC:MIC ratio target associated with net bacterial stasis from a neutropenic murine-thigh infection model (16). The latter endpoint is considered a reasonable non-clinical endpoint for the assessment for ABSSSI (5, 21) and, as described above, is supported by the results of the clinical PK-PD analyses undertaken for omadacycline using data from the OASIS-1 and OASIS-2 studies. It is important to remember that the assessment of PK-PD target attainment based on the evaluation of a non-clinical PK-PD target for efficacy represents an intermediary assessment. That is, it represents the probability of achieving a drug exposure associated with efficacy in a non-clinical infection model, not the probability of a clinical response in a patient population. As such, predictions based on these data can be expected to be directionally informative. In contrast, the above-described clinical PK-PD relationships for efficacy were influenced by the underlying patient immune function and comorbidities and, accordingly, were considered more relevant when assessing efficacy by MIC value. Thus, while observed efficacy and percent probabilities of PK-PD target attainment by MIC were considered, greater emphasis was placed on the results of percent probabilities of model-predicted success for ECR by MIC.

At an MIC value of 0.25 µg/mL, the 95% lower confidence bounds for the probability of model-predicted success for ECR for the IV-to-PO and PO-only dosing regimens were near 90%. These 95% lower confidence bounds for the percent probabilities of success for ECR at an MIC value of 0.25 µg/mL were substantially higher than a roughly 80% success likelihood estimated by the models for patients with no treatment (via the intercept of the continuous relationship) or the observed success in the lower group for the two-group relationship. At an MIC value of 0.5 µg/mL, model-predicted probabilities of success for ECR were near 90% for both dosing regimens, with 95% lower confidence bounds near 80%. However, at an MIC value of 1 µg/mL, the model-predicted probabilities of success for ECR were above 80% for both dosing regimens, but the 95% lower confidence bounds were substantially below 80% for both dosing regimens. In addition to the reduced percent probabilities of success for ECR at an MIC value of 1 µg/mL, there was additional uncertainty at this MIC value since there were no patients with ABSSSI and *S. aureus* at baseline and PK data who had an MIC value above 0.5 µg/mL in the PK-PD analysis data set. Thus, the observed clinical data were too limited to provide support for extrapolations of the PK-PD relationship to an MIC value of 1 µg/mL. The poor estimated results and their uncertainty at an MIC value of 1 µg/mL, however, were consistent with the omadacycline susceptibility breakpoint for *S. aureus* of 0.5 µg/mL based on the US FDA interpretive criteria (22).

When comparing the percent probabilities of model-predicted success for ECR to the observed outcomes by MIC value, it is important to note that the observed outcomes did not demonstrate discernable trends with increased or decreased MIC in the micro-mITT and ME populations inclusive of patients without PK data. The absence of such relationships by MIC alone suggests the benefit of using an index that incorporates the AUC in addition to the MIC. This benefit was also demonstrated by the results of the PK-PD analyses for ECR, for which some evidence of relationships with each of the free-drug plasma AUC:MIC ratio and AUC was observed but for which there was no evidence of a relationship with MIC. The evaluation of free-drug plasma AUC:MIC ratio as a continuous variable had an added benefit of providing greater statistical power for the identification of relationships than the evaluation of MIC, which had only a small number of distinct observed values.

In conclusion, PK-PD analyses for efficacy using data from omadacycline-treated patients with ABSSSI enrolled in the OASIS-1 (IV-to-PO) and OASIS-2 (PO-only) studies (10, 11) demonstrated relationships between ECR and free-drug plasma AUC:MIC ratio evaluated in multiple forms. Based on these relationships, the lowest response was 80% for patients with a free-drug plasma AUC:MIC ratio below 12.5. The application of these PK-PD relationships among simulated patients demonstrated that percent probabilities of model-predicted success for ECR exceeded or approached 90% at an MIC value of 0.5 µg/mL. Thus, the findings of the PK-PD analyses provided support for omadacycline IV-to-PO and PO-only dosing regimens for ABSSSI and the current FDA omadacycline susceptibility breakpoint for *S. aureus* of 0.5 µg/mL. Lastly and, perhaps, the most important for future drug development programs for ABSSSI, the results of these analyses also serve to demonstrate that bacterial reductions greater than net bacterial stasis may be larger than necessary to predict high probabilities of success for ECR in patients with ABSSSI.

## MATERIALS AND METHODS

### PK-PD analyses for efficacy

#### Study data

Data from omadacycline-treated patients with ABSSSI were collected from two phase 3 clinical studies, OASIS-1 and OASIS-2 (10, 11). OASIS-1 was a randomized (1:1) active comparator-controlled, double-blind, multi-center study of IV and PO omadacycline compared to IV and PO linezolid for the treatment of adult subjects with ABSSSI known or suspected to be due to Gram-positive pathogens (10). Patients enrolled in OASIS-1 were randomized to receive omadacycline 100 mg IV q12h for two doses, followed by 100 mg IV q24h, with the option to switch to 300 mg PO q24h after 3 days, or linezolid 600 mg IV q12h with the option to switch to 600 mg PO q12h after 3 days (10). OASIS-2 was a randomized (1:1), double-blind, double-dummy, active comparator-controlled, multi-center study comparing omadacycline PO and linezolid PO for the treatment of adult subjects with ABSSSI known or suspected to be due to Gram-positive pathogens. Patients enrolled in OASIS-2 were randomized to receive omadacycline 450 mg PO q24h for two doses, followed by 300 mg PO q24h or linezolid 600 mg PO q12h (11). The duration of treatment for both studies was 7–14 days. Blood samples for the assay of omadacycline were collected during therapy from patients enrolled in OASIS-1 and OASIS-2. The sampling schedule for each study was previously described (13). The efficacy endpoints assessed among patients enrolled in these two studies are described below (10, 11, 23).

The analysis population for the PK-PD analyses consisted of patients with PK data from the microbiologically evaluable populations from each study and who had *S. aureus* isolated from cultures obtained at baseline.

Clinical response was evaluated at 48–72 hours, EOT, and PTE (7–14 days after EOT). Success for ECR at 48–72 hours was defined as survival with the size of the primary lesion reduced by ≥20% relative to baseline without receiving any rescue antibacterial therapy. Investigator-assessed clinical response was evaluated at EOT and PTE, with success defined as survival with the infection sufficiently resolved, and without receiving any rescue antibacterial therapy or development of an adverse event that required discontinuation. Investigator-assessed overall clinical response at PTE was based on the results of investigator-assessed clinical responses at EOT and PTE.

Patient-level microbiological response was evaluated at EOT and PTE, with success defined as eradication or presumed eradication based on investigator-assessed clinical response in the absence of material available for culture. Patient-level overall microbiological response at PTE was based on the results of the patient-level microbiological responses at EOT and PTE.

Lesion size was assessed serially on days 2–7, day 10, EOT, and PTE. Changes in lesion size at each time point relative to baseline were assessed as continuous efficacy endpoints. Achievement of cessation of spread and reduction thresholds of 10, 20, 30, 50, and 70% at individual time points were assessed as dichotomous efficacy endpoints. For each percent reduction threshold, the time to achievement was assessed as a time-to-event efficacy endpoint.

#### Population pharmacokinetic model

A previously developed population PK model based on IV and PO PK data from phase 1 and 3 studies (13) was used to generate omadacycline AUC values among patients from the OASIS-1 and OASIS-2 studies (10, 11) and simulated patients. A brief description of this population PK model is described below.

Data used to develop the population PK model were from 13 phase 1 studies, a phase 1b uncomplicated urinary tract infection study (24), one phase 3 CABP study (9), and two phase 3 ABSSSI studies, one of which included the OASIS-1 study (8, 10). This model was subsequently assessed using data from the OASIS-2 study (11). The final population PK model was a linear, three-compartment model with zero-order IV input or first-order absorption using transit compartments to account for absorption delay following oral dosing. Evaluation of the effects of covariates on PK demonstrated that sex was a significant covariate on multiple PK parameters. However, the net effect of sex on the omadacycline concentration-time profile was found to be minimal.

The robust fit of the model to the plasma data suggested that individual exposures in omadacycline-treated patients were sufficiently accurate and precise, criteria which are important for conducting PK-PD analyses herein. Additionally, results of the above-described assessment to qualify the population PK model using PK data from OASIS-2 (11) demonstrated that the predictive performance of the model was robust, further increasing the confidence in individual predicted AUC values for the PK-PD analyses for efficacy based on data from patients in both the OASIS-1 and OASIS-2 studies (10, 11).

#### Univariable and multivariable PK-PD analyses

Using the above-described population PK model (13) and the dosing history and Bayesian post-hoc PK parameter estimates for each patient evaluated for the PK-PD analyses for efficacy, a PK simulation was performed using the mrgsolve package in R version 3.3.1 (25, 26) to generate individual omadacycline total-drug plasma concentration-time profiles from 0 to 48 hours for patients from the OASIS-1 and OASIS-2 studies (10, 11) included in the analysis population for the PK-PD studies. Average 24-hour omadacycline total-drug plasma AUC values were calculated by numerical integration of the total-drug plasma concentration-time profiles from 0 to 48 hours and then dividing the resulting AUC value by 2. Using a protein binding estimate of 21% based on *in vitro* data for human plasma protein binding, total-drug plasma AUC values were adjusted to free-drug plasma AUC values using a free fraction of 0.79 (27).

Relationships between efficacy endpoints described in Table S8 and free-drug plasma AUC:MIC ratio were evaluated. The data supporting the choice of this PK-PD index for omadacycline efficacy were based on the data from other tetracycline agents that demonstrated that free-drug plasma AUC:MIC ratio was the PK-PD index predictive of efficacy (17–20). The omadacycline MIC value of the baseline infecting pathogen was used to calculate free-drug plasma AUC:MIC ratio.

Univariable relationships between efficacy endpoints and free-drug plasma AUC:MIC ratio were evaluated using the following procedures implemented in R version 3.3.1 (26): Chi-square tests, Fisher’s exact tests, and/or logistic regression for dichotomous endpoints (clinical and microbiological response and lesion size reduction threshold endpoints); Kruskal-Wallis tests and Spearman correlations for continuous endpoints (for percent reduction in lesion size by day endpoints); and log-rank tests and Cox proportional hazards regression for time-to-lesion size reduction endpoints. In addition to assessments of the continuous form of free-drug plasma AUC:MIC ratio, two- and three-group categorical forms of free-drug plasma AUC:MIC ratio were assessed using thresholds optimized for statistical significance. Multivariable analyses were considered for any efficacy endpoint for which a biologically plausible univariable relationship was identified at a 0.10 significance level (i.e., *P* ≤ 0.10) and for which there were a sufficient number of failures in the case of dichotomous efficacy endpoints. Biologically plausible univariable relationships were those for which increased free-drug plasma AUC:MIC ratio was associated with improved response. Relationships between the efficacy endpoints and each of AUC and MIC individually were also assessed.

If univariable relationships were found between efficacy endpoints and free-drug plasma AUC:MIC ratio evaluated as a categorical variable, then assessments of the optimized thresholds were considered. In particular, magnitudes of such thresholds and their 95% bootstrap confidence intervals were compared to non-clinical free-drug plasma AUC:MIC ratio targets for omadacycline efficacy determined using data for 10 *S. aureus* isolates evaluated in a neutropenic murine-thigh infection model (16). If relationships between efficacy endpoints and free-drug plasma AUC:MIC ratio evaluated as a continuous variable were identified using logistic regression, then predicted probabilities of success at values of non-clinical PK-PD targets and optimized grouping thresholds for free-drug plasma AUC:MIC ratio were estimated using the resulting models.

### Evaluation of omadacycline dosing regimens

#### Monte Carlo simulations

Using the above-described population PK model (13) and the covariate, sex, which was assigned to simulated patients in equal proportions, individual post-hoc parameter estimates were generated for 5,000 simulated patients using the mrgsolve package in R version 3.3.1 (25, 26 ). Using the above-described post-hoc parameter estimates, total-drug plasma concentration-time profiles from 0 to 120 hours were generated for each simulated patient after administration of the following two IV-to-PO and one PO-only omadacycline dosing regimens: (i) 100 mg IV q12h on day 1, followed by 100 mg IV q24h on day 2 and 300 mg PO q24h on days 3–5; (ii) 200 mg IV q24h on day 1, followed by 100 mg IV on day 2 and 300 mg PO q24h on days 3–5; and (iii) 450 mg PO q24h on days 1–2, followed by 300 mg PO q24h on days 3–5.

Average 24-hour total-drug plasma AUC values were calculated by numerical integration of the total-drug plasma concentration curves from 0 to 48 hours and then dividing the resulting AUC by 2. Additionally, 24-hour total-drug plasma AUC values were determined after the PO switch on day 3 or 5 for IV-to-PO dosing regimens. Total-drug plasma AUC values were adjusted to free-drug plasma AUC values using the above-described free fraction of 0.79 for human plasma protein binding (27).

#### Evaluation of PK-PD target attainment by MIC among simulated patients

Non-clinical PK-PD targets for omadacycline efficacy were determined using data from a neutropenic murine-thigh infection model in which 10 *S. aureus* isolates were evaluated (16). The range of omadacycline MIC values for these *S. aureus* isolates was 0.25–0.5 µg/mL. As shown in Table S9, the median (minimum, maximum) free-drug plasma AUC:MIC ratio target associated with net bacterial stasis for *S. aureus* was 21.9 (13.8, 51.1).

Free-drug plasma AUC:MIC ratio targets were randomly assigned for a simulated patient based on an estimated log normal distribution of targets associated with net bacterial stasis. The basis for evaluating randomly assigned free-drug plasma AUC:MIC ratio targets has been described previously (28). The log normal distribution of free-drug plasma AUC:MIC ratio targets was truncated at ±2 standard deviations on the log scale.

This choice to evaluate free-drug plasma AUC:MIC ratio targets associated with net bacterial stasis was supported by results of previous PK-PD analyses based on clinical data which demonstrated that achieving a PK-PD target associated with net bacterial stasis was associated with a high percentage of successful outcomes in patients with ABSSSI (21).

#### Evaluation of PK-PD model-predicted efficacy by MIC among simulated patients

Using relationships between a given efficacy endpoint and free-drug plasma AUC:MIC ratio, model-predicted percent probability of achieving a successful outcome was determined at MIC values for each simulated patient after administration of omadacycline dosing regimens. Predicted percent probabilities of response were averaged across simulated patients by MIC value for each dosing regimen. For any relationships identified between dichotomous efficacy endpoints and the continuous form of free-drug plasma AUC:MIC ratio, 300 bootstrap samples were used to construct a 95% lower confidence bound for the model-predicted percent probability of achieving a successful response at individual MIC values.

#### Assessments of overall PK-PD target attainment and model-predicted efficacy averaged over MIC distributions among simulated patients

Percent probabilities of PK-PD target attainment on days 1–2 and mean percent probabilities of response by MIC were evaluated relative to *S. aureus* MIC distributions for isolates collected from the USA and Europe (1). A total of 4,215 *S*. *aureus* [1,438 methicillin-resistant *S. aureus* (MRSA) and 2,777 methicillin-susceptible *S. aureus* (MSSA)] isolates with omadacycline MIC values that ranged from ≤0.015 to 8 µg/mL, 0.03 to 8 µg/mL, and ≤0.015 to 4 µg/mL for all isolates, and the MRSA and MSSA isolate subsets, respectively, were collected. Omadacycline MIC values at which 50 and 90% of all isolates in a collection were inhibited were 0.12 and 0.25 µg/mL, respectively, among both the MRSA and MSSA subsets.

Using the MIC distributions for all isolates pooled and stratified by MRSA and MSSA, overall percent probabilities of PK-PD target attainment (i.e., the weighted average) were determined. In addition, if a meaningful clinical PK-PD relationship for efficacy was identified, overall average percent probabilities of model-predicted response for such an efficacy endpoint were determined using the above-described MIC distributions.

## SUPPLEMENTAL MATERIAL

Supplemental tables and figure aac.01281-23-s0001.docx Tables S1 to S9 and Fig. S1.

ASM does not own the copyrights to Supplemental Material that may be linked to, or accessed through, an article. The authors have granted ASM a non-exclusive, world-wide license to publish the Supplemental Material files. Please contact the corresponding author directly for reuse.
