# Supplementary material for: Assessment of pharmacokinetics-pharmacodynamics to support omadacycline dosing regimens for the treatment of patients with acute bacterial skin and skin structure infections
Source: Antimicrob Agents Chemother. 2024 Jul 31;68(9):e01281-23. doi: 10.1128/aac.01281-23 (PMC11373226; doi:10.1128/aac.01281-23)
Supplement: Supplemental tables and figure — Tables S1 to S9 and Fig. S1. [file aac.01281-23-s0001.docx]

| **Table S1**. Summary statistics for categorical patient characteristics for patients with *S. aureus* | | | |
| --- | --- | --- | --- |
| **Baseline characteristic** | **% (n/N) of patients with *S. aureus* by study and pooled** | | |
|  | **OASIS-1 (N=31)** | **OASIS-2 (N=97)** | **Pooled**  **(N=128)** |
| Race |  |  |  |
| White | 87.1 (27/31) | 89.7 (87/97) | 89.1 (114/128) |
| Black or African American | 12.9 (4/31) | 4.1 (4/97) | 6.2 (8/128) |
| Asian | 0 (0/31) | 1.0 (1/97) | 0.8 (1/128) |
| American Indian or Alaska Native | 0 (0/31) | 2.1 (2/97) | 1.6 (2/128) |
| Other | 0 (0/31) | 3.1 (3/97) | 2.3 (3/128) |
| Sex (male) | 67.7 (21/31) | 71.1 (69/97) | 70.3 (90/128) |
| Infection type |  |  |  |
| Cellulitis/erysipelas | 51.6 (16/31) | 11.3 (11/97) | 21.1 (27/128) |
| Major cutaneous abscess | 35.5 (11/31) | 16.5 (16/97) | 21.1 (27/128) |
| Wound infection | 12.9 (4/31) | 72.2 (70/97) | 57.8 (74/128) |
| MRSA | 45.2 (14/31) | 49.5 (48/97) | 48.4 (62/128) |
| Polymicrobial infection | 19.4 (6/31) | 37.1 (36/97) | 32.8 (42/128) |
| Gram-negative pathogen at baseline | 9.7 (3/31) | 8.2 (8/97) | 8.6 (11/128) |
| Presence of diabetes | 9.7 (3/31) | 4.1 (4/97) | 5.5 (7/128) |
| IV drug abuse^a^ | 29.0 (9/31) | 73.2 (71/97) | 62.5 (80/128) |
| MIC (µg/mL) |  |  |  |
| 0.12 | 16.1 (5/31) | 6.2 (6/97) | 8.6 (11/128) |
| 0.25 | 54.8 (17/31) | 73.2 (71/97) | 68.8 (88/128) |
| 0.5 | 29.0 (9/31) | 20.6 (20/97) | 22.7 (29/128) |
| a. Represents patients who injected drugs or had infection due to injection drug use. | | | |

| **Table S2.** Summary statistics for continuous patient characteristics for patients with *S. aureus* | | | | | | |
| --- | --- | --- | --- | --- | --- | --- |
| **Baseline characteristic** | **OASIS-1 (N=31)** | | **OASIS-2 (N=97)** | | **Pooled (N=128)** | |
|  | **Mean (%CV)** | **Median (Min, Max)** | **Mean (%CV)** | **Median (Min, Max)** | **Mean (%CV)** | **Median (Min, Max)** |
| Age (yr) | 51.9 (32.5) | 51  (24, 88) | 42.7 (26.7) | 42  (20, 69) | 44.9 (29.9) | 43  (20, 88) |
| BMI (kg/m^2^) | 28.5 (18.4) | 27.9  (20.9, 46.6) | 27.2 (21.7) | 26.5  (16.3, 56.6) | 27.5 (20.9) | 26.7  (16.3, 56.6) |
| CLcr (mL/min/1.73 m^2^) | 84.4 (45.7) | 79.5  (23.1, 169) | 106 (31.3) | 104  (39.0, 214) | 101 (35.4) | 99.7  (23.1, 214) |
| Height (cm) | 173 (5.70) | 175  (153, 201) | 172 (5.00) | 172  (156, 194) | 172 (5.2) | 173  (153, 201) |
| Weight (kg) | 85.7 (21.7) | 83.1 (60.0, 147) | 80.6 (21.3) | 79.8  (41.7, 164) | 81.8 (21.5) | 80.7  (41.7, 164) |

| **Table S3.** Summary of successful responses for efficacy endpoints by visit for patients with *S. aureus* | | | | | |
| --- | --- | --- | --- | --- | --- |
| **Visit** | **Percentage of successful responses by efficacy endpoint and visit (n/N)** | | | | |
|  | **Early clinical response** | **Investigator-assessed clinical response** | **Investigator-assessed overall clinical response** | **Microbiological response** | **Overall microbiological response** |
| 48-72 hours | 92.8 (116/125) | - | - | - | - |
| EOT | - | 99.2 (126/127) | - | 99.2  (126/127) | - |
| PTE | - | 99.1 (114/115) | 99.1 (114/115) | 99.1  (114/115) | 99.1  (114/115) |

| **Table S4.** Summary of dichotomous lesion area endpoints by day or visit for patients with *S. aureus* | | | | | | |
| --- | --- | --- | --- | --- | --- | --- |
| **Assessment day  or visit** | **Dichotomous lesion area endpoints % (n/N)** | | | | | |
|  | **Cessation** | **≥ 10% reduction** | **≥ 20% reduction** | **≥ 30% reduction** | **≥ 50% reduction** | **≥ 70% reduction** |
| Day 2 | 96.1 (123/128) | 77.3 (99/128) | 60.2 (77/128) | 48.4 (62/128) | 17.2 (22/128) | 3.10 (4/128) |
| Day 3 | 99.2 (126/127) | 96.1 (122/127) | 90.6 (115/127) | 85.0 (108/127) | 63.0 (80/127) | 25.2 (32/127) |
| Day 4 | 100 (31/31) | 96.8 (30/31) | 93.5 (29/31) | 90.3 (28/31) | 64.5 (20/31) | 38.7 (12/31) |
| Day 5 | 100 (31/31) | 96.8 (30/31) | 96.8 (30/31) | 93.5 (29/31) | 83.9 (26/31) | 67.7 (21/31) |
| Day 6 | 100 (17/17) | 94.1 (16/17) | 94.1 (16/17) | 94.1 (16/17) | 88.2 (15/17) | 64.7 (11/17) |
| Day 7 | 100 (82/82) | 100 (82/82) | 98.8 (81/82) | 97.6 (80/82) | 93.9 (77/82) | 84.1 (69/82) |
| Day 10 | 100 (19/19) | 100 (19/19) | 100 (19/19) | 100 (19/19) | 100 (19/19) | 94.7 (18/19) |
| EOT | 100 (128/128) | 100 (128/128) | 100 (128/128) | 99.2 (127/128) | 99.2 (127/128) | 98.4 (126/128) |
| PTE | 100 (123/123) | 100 (123/123) | 100 (123/123) | 100 (123/123) | 100 (123/123) | 99.2 (122/123) |

| **Table S5.** Summary statistics for omadacycline free-drug plasma AUC, baseline MIC, and free-drug plasma AUC:MIC ratio for patients with *S. aureus* | | | | | | | | | |
| --- | --- | --- | --- | --- | --- | --- | --- | --- | --- |
| **Measure** | **OASIS-1** **(N=31)** | | | **OASIS-2 (N=97)** | | | **Pooled (N=128)** | | |
|  | **Free-drug plasma AUC^a^ (mg•h/L)** | **Baseline MIC (µg/mL)** | **Free-drug plasma AUC:MIC ratio^a^** | **Free-drug plasma AUC^a^ (mg•h/L)** | **MIC (µg/mL)** | **Free-drug plasma AUC:MIC ratio^a^** | **Free-drug plasma AUC^a^ (mg•h/L)** | **Baseline MIC (µg/mL)** | **Free-drug plasma AUC:MIC ratio^a^** |
| Mean (%CV) | 10.3  (22.2) | - | 41.6  (51.9) | 5.60  (42.0) | - | 21.6  (62.7) | 6.74  (45.8) | - | 26.4  (68.0) |
| Median or MIC_50/90_ (Min, Max) | 9.75  (7.08, 16.7) | 0.25, 0.5  (0.12, 0.5) | 36.8  (15.4, 98.4) | 5.27  (0.79, 11.6) | 0.25, 0.5  (0.12, 0.5) | 19.6  (3.15, 95.3) | 6.42  (0.79, 16.7) | 0.25, 0.5  (0.12, 0.5) | 22.8  (3.15, 98.4) |
| - 1. a. Based on the 24-hour average AUC over 0 to 48 hours. | | | | | | | | | |

| **Table S6.** Summary of p-values for univariable relationships between the probability of achieving dichotomous efficacy endpoints and free-drug plasma AUC:MIC ratio based on data from patients with *S. aureus* | | | | |
| --- | --- | --- | --- | --- |
| **Form of independent variable for  free-drug plasma AUC:MIC ratio^a^** | **Efficacy endpoint** | **P-value by visit^b^** | | |
|  |  | **48-72 hours** | **EOT** | **PTE** |
| Continuous | ECR | 0.07  (/) | - | - |
|  | Investigator-assessed clinical response | - | 0.95 | 0.92 |
|  | Investigator-assessed overall clinical response | - | - | 0.92 |
|  | Microbiological response | - | 0.95 | 0.92 |
|  | Overall microbiological response | - | - | 0.92 |
| Quartiles | ECR | 0.31 | - | - |
|  | Investigator-assessed clinical response | - | 0.24 | 0.24 |
|  | Investigator-assessed overall clinical response | - | - | 0.24 |
|  | Microbiological response | - | 0.24 | 0.24 |
|  | Overall microbiological response | - | - | 0.24 |
| Three-group | ECR | 0.013  (//) | - | - |
|  | Investigator-assessed clinical response | - | 0.58 | 0.30 |
|  | Investigator-assessed overall clinical response | - | - | 0.30 |
|  | Microbiological response | - | 0.58 | 0.30 |
|  | Overall microbiological response | - | - | 0.30 |
| Two-group | ECR | 0.016  (/) | - | - |
|  | Investigator-assessed clinical response | - | 0.43 | 0.45 |
|  | Investigator-assessed overall clinical response | - | - | 0.45 |
|  | Microbiological response | - | 0.43 | 0.45 |
|  | Overall microbiological response | - | - | 0.45 |
| 1. AUC:MIC ratio was calculated using the average 24-hour AUC calculated over 0 to 48 hours. 2. The symbol in parenthesis after the p-value represents the direction of the univariable relationship which is provided for relationships for which p ≤ 0.1. A “/” represented increased response as AUC:MIC ratio increased. A “//” represented increased response across both pairs of adjacent segments of the three-group form of the AUC:MIC ratio. | | | | |

| **Table S7**. Comparison of observed percentages of successful response by MIC among patients with ABSSSI and *S. aureus* at baseline from the pooled Phase 3 OASIS-1 and OASIS-2 studies and percent probabilities of PK-PD target attainment on Days 1 to 2 and model-predicted success for ECR by MIC among simulated patients after the administration of omadacycline IV-to-PO dosing regimens, with 100 mg IV q12h and 200 mg IV q24h loading doses on Day 1 | | | | | | | | | | |
| --- | --- | --- | --- | --- | --- | --- | --- | --- | --- | --- |
| **MIC (µg/mL)** | **Observed percentage of successful response  by MIC by study population^a^** | | | | **Percent probability of PK-PD target attainment on Days 1 to 2 and model-predicted success for ECR at 48 to 72 hours for two univariable PK-PD relationships by MIC among simulated patients^b, c^ by omadacycline dosing regimen** | | | | | |
|  |  |  |  |  | **Assessment of IV-to-PO dosing regimen with 100 mg IV q12h loading doses on Day 1^d^** | | | **Assessment of IV-to-PO dosing regimen  with a 200 mg IV q24h loading dose on Day 1^e^** | | |
|  | **micro-mITT population** | | **ME populations** | | **PK-PD target attainment^f^** | **Univariable PK-PD relationships** | | **PK-PD target attainment^f^** | **Univariable PK-PD relationships** | |
|  | **ECR at**  **48 to 72 hours (N=365)** | **Clinical response at PTE (N=365)** | **ECR at**  **48 to 72 hours (N=339)** | **Clinical response  at PTE (N=291)** |  | **Continuous** | **Two-Group** |  | **Continuous** | **Two-Group** |
| 0.06 | 0 | 0 | 0 | 0 | 100 | 100 | 96.0 | 100 | 100 | 96.0 |
| 0.12 | 92.7  (38/41) | 80.5  (33/41) | 97.4  (38/39) | 100  (33/33) | 100 | 99.5 | 96.0 | 100 | 99.6 | 96.0 |
| 0.25 | 86.5 (225/260) | 81.9 (213/260) | 93.3 (224/240) | 97.5 (199/204) | 91.3 | 96.8 | 96.0 | 92.6 | 97.0 | 96.0 |
| 0.5 | 91.9  (57/62) | 90.3  (56/62) | 96.6  (56/58) | 100  (53/53) | 35.1 | 91.9 | 95.6 | 38.0 | 92.1 | 95.7 |
| 1 | 100 (2/2) | 100 (2/2) | 100 (2/2) | 100  (1/1) | 1.04 | 87.2 | 81.5 | 1.40 | 87.4 | 81.9 |
| **Overall^g^** | 88.2 (322/365) | 83.3  (304/365) | 94.4  (320/339) | 98.3  (286/291) |  |  |  |  |  |  |
| All |  |  |  |  | 95.8 | 98.9 | 95.8 | 96.1 | 98.9 | 95.8 |
| MRSA |  |  |  |  | 92.3 | 98.4 | 95.4 | 92.7 | 98.5 | 95.4 |
| MSSA |  |  |  |  | 97.7 | 99.1 | 96.0 | 97.9 | 99.2 | 96.0 |
| 1. Based on data from patients with ABSSSI and *S. aureus* at baseline in the micro-mITT and ME populations of the OASIS-1 and OASIS-2 studies [1, 2]. 2. Assessed using free-drug plasma AUC:MIC ratio targets associated with a net bacterial stasis CFU reduction from baseline for *S. aureus* based on data from a neutropenic murine-thigh infection model [3]. 3. Based on the assessment of average free-drug plasma AUC_0-24_ values on Days 1 and 2. 4. Omadacycline 100 mg IV q12h on Day 1, followed by 100 mg IV q24h on Day 2 with a PO switch to 300 mg PO q24h on Days 3 to 5. 5. Omadacycline 200 mg IV q24h on Day 1, followed by 100 mg IV q24h on Day 2 and 300 mg PO q24h on Days 3 to 5. 6. Using data for all *S. aureus* isolates studied, free-drug plasma AUC:MIC ratio targets associated with net bacterial stasis were randomly assigned based on an estimated log normal distribution of AUC:MIC ratio targets associated with the same endpoint. 7. Overall represents the percentage of successful clinical response among all observed patients or the percent probability of PK-PD target attainment weighted over the given MIC distribution [4] for simulated patients. | | | | | | | | | | |

| **Table S8.** Summary of efficacy endpoints assessed | | |
| --- | --- | --- |
| Efficacy endpoint | Efficacy endpoint type | Evaluation time point |
| Early clinical response^a^ | Dichotomous | 48 to 72 hours after the first dose of test article |
| Investigator-assessed clinical response^a^ | Dichotomous | EOT and/or PTE |
| Investigator-assessed overall clinical response^a^ | Dichotomous | PTE |
| Microbiological response^b,c^ | Dichotomous | EOT, PTE |
| Overall microbiologic response^b,c^ | Dichotomous | PTE |
| Change in lesion size relative to baseline | Dichotomous, time-to-event, and continuous | Screening then Days 2-10, EOT and PTE |
| 1. Patients categorized as a clinical success included those with responses of success while patients categorized as a clinical failure included those with responses of failure. Patients with indeterminate clinical response were excluded from the analyses. Additionally, patients for whom failure was declared for clinical or overall clinical response for a given visit was not due to study drug (e.g., due to an adverse event, lost to follow up, or death occurred to a non-infection related cause) were also excluded. Accordingly, if failure was declared for microbiological or overall microbiological response in such patients for whom clinical failure was excluded at the same visit, these responses were also excluded for such patients. 2. Microbiological and overall microbiological response were assessed at the patient-level. 3. Patients categorized as a microbiological success included those with responses of eradication or presumed eradication, while patients categorized as a microbiological failure included those with a response of persistence or presumed persistence. | | |

| **Table S9.** Omadacycline free-drug plasma AUC:MIC ratio targets for *S. aureus* efficacy based on data from a neutropenic murine-thigh infection model | | | | |
| --- | --- | --- | --- | --- |
| **Isolate** | **MIC  (µg/mL)** | **Free-drug plasma AUC:MIC ratio targets by bacterial reduction endpoint** | | |
|  |  | **Net bacterial stasis** | **1-log_10_ CFU reduction from baseline** | |
| ATCC 29213 | 0.25 | 29.6 | 58.8 | |
| SMITH | 0.25 | 51.1 | 302.5 | |
| MW2 | 0.5 | 23.1 | 52.5 | |
| R2527 | 0.5 | 21.7 | 62.1 | |
| 6538P | 0.25 | 22.1 | 49.0 | |
| ATCC 25923 | 0.25 | 22.7 | 61.6 | |
| ATCC 33591 | 0.5 | 16.2 | 56.6 | |
| WIS-1 | 0.5 | 13.8 | 42.5 | |
| LSI 1848 | 0.5 | 20.4 | 62.9 | |
| 307109 | 0.5 | 16.5 | 32.2 | |
| Mean (SD) | ̶ | 23.7 (10.6) | 78.1 (79.5) | |
| Median | ̶ | 21.9 | 57.7 | |
| 1. Based on data from studies conducted by Lekpak *et al*. [3]. | | | |  |

**Figure S1.** Percent probabilities of PK-PD target attainment on Days 1 to 2 and model-predicted success for ECR by MIC among simulated patients after administration of omadacycline IV-to-PO dosing regimens with 100 mg IV q12h (A) and 200 mg IV q24h (B) loading doses on Day 1, overlaid on MIC distributions for *S. aureus* isolates from the USA and Europe

**
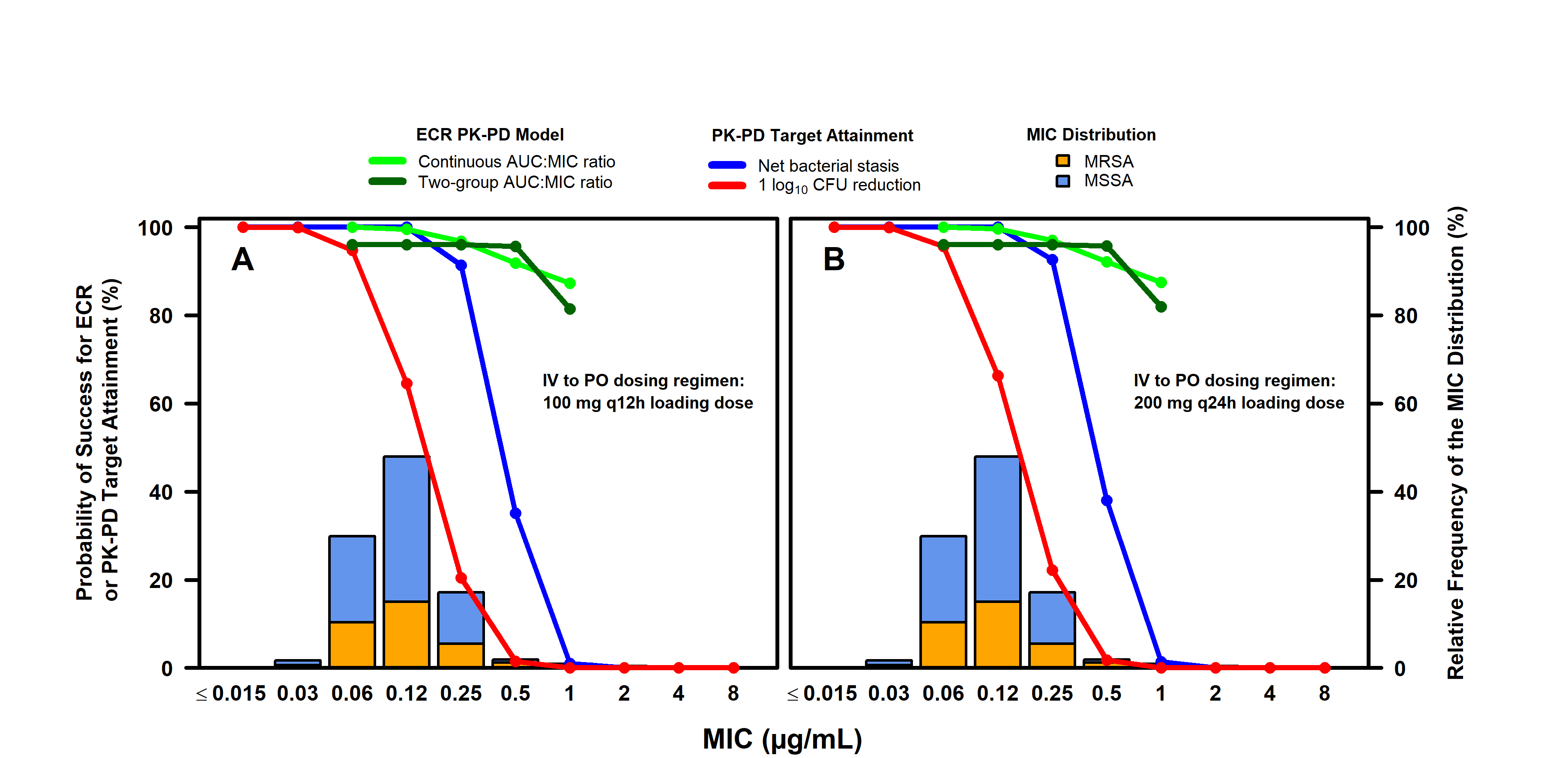
**

**References**

1. O’Riordan W, Green S, Overcash JS, Puljiz I, Metallidis S, Gardovskis J, Garrity-Ryan L, Das AF, Tzanis E, Eckburg PB, Manley A, Villano SA, Steenbergen JN, Loh E. 2019. Omadacycline for acute bacterial skin and skin-structure infections. N Engl J Med 380:528-538.
2. O’Riordan W, Cardenas C, Shin E, Sirbu A, Garrity-Ryan L, Das AF, Eckburg PB, Manley A, Steenbergen JN, Tzanis E, McGovern PC, Loh E. 2019. Once-daily oral omadacycline versus twice-daily oral linezolid for acute bacterial skin and skin structure infections (OASIS-2): a Phase 3, double-blind, multicentre, randomised, controlled, non-inferiority trial. Lancet Infect Dis 19:1080-1090. DOI: 10.1016/S1473-3099(19)30275-0
3. Lepak AJ, Zhao M, Marchillo K, VanHecker J, Andes DR. 2019. *In vivo* pharmacodynamics of omadacycline against *Staphylococcus aureus* in the neutropenic murine thigh infection model. Antimicrob Agents Chemother 63:e00624-19.
4. Pfaller MA, Huband MD, Shortridge D, Flamm RK. 2018. Surveillance of omadacycline activity tested against clinical isolates from the United States and Europe as part of the 2016 SENTRY antimicrobial surveillance program. Antimicrob Agents Chemother 62:e02327-17.
